# Malaria after liver transplantation: Report of two cases and a review of published cases

**DOI:** 10.7705/biomedica.7433

**Published:** 2025-05-30

**Authors:** Andrés Fernando Rodríguez-Gutiérrez,, Isabel Cristina Ramírez-Sánchez

**Affiliations:** 1 Grupo de Gastrohepatología, Universidad de Antioquia, Medellín, Colombia Universidad de Antioquia Universidad de Antioquia Medellín Colombia; 2 Departamento de Medicina Interna, Universidad de Antioquia, Medellín, Colombia Universidad de Antioquia Universidad de Antioquia Medellín Colombia; 3 Servicio de Infectología, Hospital Pablo Tobón Uribe, Medellín, Colombia Hospital Pablo Tobón Uribe Medellín Colombia

**Keywords:** Plasmodium, Malaria, liver transplantation, Colombia, Plasmodium, malaria, trasplante de hígado, Colombia

## Abstract

Malaria is a tropical disease that is rarely reported in liver transplant recipients. However, some cases have been documented around the world and here we report two.

*Case 1*. A 54-year-old male attended the emergency room 30 days after liver transplantation due to malaise, fever, chills, thrombocytopenia, and anemia. *Plasmodium vivax* was detected in the blood smear, and the patient was treated with artemether/ lumefantrine, achieving resolution of the parasitemia. Neither chloroquine nor primaquine were prescribed because they were unavailable in the country. Two months later, the patient returned to the emergency room with the same symptoms and was diagnosed with a relapse of malaria caused by *P. vivax*. The patient received successful treatment with chloroquine and primaquine, which were already available. Given that the liver donor came from a malaria-endemic area, the infection was probably of donor origin, likely by hypnozoites present in the allograft.

*Case 2*. A 58-year-old woman living in a malaria-endemic region attended the emergency service with fever, malaise, arthralgia, cytopenias, and hypertransaminasemia six months after undergoing a liver transplant. *P. vivax* was detected in the blood smear, so treatment with chloroquine and primaquine was started. After treatment, the blood smear was negative, and the patient was discharged. In this case, the infection was likely caused by a vector bite in its endemic area of residence or remotely derived from the graft, but it occurred six months after the procedure.

The two cases described here add to the 15 cases of malaria in liver transplant recipients that have been reported in the world. Most of the cases occurred within the first two months after the transplantation, and the outcome was usually favorable, nevertheless, early detection and treatment are essential.

## Resumen

La malaria es una enfermedad tropical que no se observa usualmente después de un trasplante hepático. Sin embargo, hay casos documentados alrededor del mundo y en este estudio se reportan dos:

*Caso 1*. Se trata de un hombre de 54 años, que consultó en enero del 2023 -30 días después de trasplante hepático- por fiebre, escalofríos, trombocitopenia y anemia. Se detectaron trofozoítos de *Plasmodium vivax* en la muestra de gota gruesa, y el paciente fue tratado con artemeter-lumefantrina, debido al desabastecimiento nacional de cloroquina y primaquina. Aunque se logró resolver la parasitemia, dos meses después reingresó por síntomas similares y se le diagnosticó una recaída de malaria por *P. vivax*. Esta vez, recibió tratamiento con cloroquina y 14 días de primaquina. A los cuatro días, el paciente fue dado de alta. Dada la procedencia del donante -zona endémica de malaria- se concluyó que la infección del paciente se derivó del donante debido a la presencia de hipnozoítos en el injerto hepático.

*Caso 2*. Se trata de una mujer de 58 años, residente en una zona endémica para malaria, que consultó en abril del 2016, seis meses después del trasplante hepático, por fiebre, malestar, artralgias, citopenias e hipertransaminasemia. Como se detectaron trofozoítos de *P. vivax* en el extendido de sangre periférica, la paciente recibió el tratamiento convencional con cloroquina y primaquina, con el que logró eliminar la parasitemia y tras el cual se le dio el alta hospitalaria. En este caso, se consideró que la infección probablemente fue causada por la picadura de un vector en el sitio de residencia, más que por el injerto, ya que la infección se presentó seis meses después del trasplante.

Los dos casos descritos se suman a los 15 casos reportados en la literatura hasta el momento. La mayoría de esos casos ocurrieron en los dos primeros meses después del trasplante y el desenlace fue favorable en general, con solo dos muertes reportadas. No obstante, la detección y el tratamiento tempranos son fundamentales.

Liver transplantation is the standard of care for patients with acute and chronic end-stage liver disease, as well as some forms of hepatocellular carcinoma ^1^. In addition, it has been reported that survival rates at one and five years after liver transplantation reach 90 and 77%, respectively ^1^.

Infections are the main complication of liver transplantation and the leading cause of death within the first year after transplant. Nonetheless, tropical diseases such as malaria should be considered in patients with liver transplantation in endemic areas or with organs from immigrant donors from those regions ^2^. However, in liver transplant recipients with malaria, the natural history of the disease, the response to immunosuppressive therapy, and the outcomes have not been fully described. Regarding these aspects, the present study reports the cases of two liver transplant patients who contracted malaria and analyzes similar cases found in PubMed and Embase.

## Case 1

A 54-year-old man residing in Medellín, Colombia (non-malaria endemic area) attended the emergency service of the *Hospital Pablo Tobón Uribe* in January 2023, a tertiary care hospital.

The patient experienced malaise, chills, and fever (up to 39°C) for four days. During the consultation, the patient reported having dysuria and tenesmus; he denied back pain and any respiratory, gastrointestinal, neurological, or skin symptoms. Thirty days before, the patient received an orthotopic liver transplantation from a deceased brain-dead donor who lived in Montería (a malaria-endemic area in northern Colombia). He had advanced cirrhosis (Child-Pugh class C; MELD score = 28) secondary to non-alcoholic steatohepatitis and alcoholic liver disease.

Before undergoing the transplant, he suffered from ascites, hydrothorax, and hepatic encephalopathy; more recently, he suffered an episode of acute- on-chronic liver failure with acute hepatorenal syndrome. Since the patient’s clinical evolution was satisfactory, he was discharged nine days after the procedure (21 days before his return to the emergency service).

Upon admission, he started immunosuppressive therapy consisting of prednisolone (20 mg once a day), azathioprine (150 mg once a day), tacrolimus (3 mg twice a day), and aspirin (100 mg once a day). Also, he was on prophylaxis with acyclovir (400 mg twice a day) and trimethoprim/ sulfamethoxazole (160 mg/800 mg three times a week). Furthermore, as the patient had tested positive for cytomegalovirus (CMV) -with a viral load of 725 copies/ml- he was on preemptive therapy (last CMV DNA test).

During the physical examination, the patient had a pulse of 105 beats per minute (bpm), blood pressure of 108/66 mm Hg, oxygen saturation of 94% (fraction of inspired oxygen: 21%), and body temperature of 38.5°C. Lung and cardiac examinations were normal, and he did not present with signs of jaundice, inflammation of the surgical wound, or abdominal pain.

The patient’s admission laboratory tests (blood chemistry and complete blood count) showed mild elevation of direct bilirubin, grade 1 acute kidney injury, anemia, thrombocytopenia, and leukopenia (table 1). The urinalysis was negative for nitrites, leukocyte esterase, leukocytes, and bacteria (with and without centrifugation). Blood and urine cultures were negative. CMV DNA viral load test was 570 copies/ml of plasma (171 IU/ml). A chest X-ray was also performed, with no abnormal findings.

**Table 1:** Results of the blood chemistry and complete blood count tests on admission in both patients.

	Case 1	Case 2
ALT (U/L)	29	77
AST (U/L)	22	165
Alkaline phosphatase (U/l)	73	156
GGT (U/l)	31.3	32
Total bilirubin (mg/dl)	1.58	1.40
Direct bilirubin (mg/dl)	1.19	0.9
Creatinine (mg/dl)	1.58	0.76
	(previous of 1.19)	
BUN (mg/dl)	43.2	15
Tacrolimus in blood (ng/ml)	8.6	No data
Hemoglobin (g/dl)	8.1	12.4
	(previous of 9.5)	
Haematocrit (%)	24	37
CRP (mg/L)	85.5	45
Leukocyte count (cel/μΙ)	5,300	1,600
		(three months ago: 6,200)
Neutrophil count (cel/μl)	4,611	1,376
Lymphocyte count (cel/μΙ)	53	192
Platelet count (cel/μΙ)	113,000	59,000
	(previous of 184,000)	(three months ago: 188,000)

ALT: Alanine aminotransferase; AST: Aspartate aminotransferase; BUN: Blood urea nitrogen; CRP: C-reactive protein; GGT: Gamma-glutamil transpeptidase

The laboratory staff informed abnormal findings in the patient’s blood tests consistent with *Plasmodium* spp. infection. Consequently, the thick blood smear showed trophozoites of *P. vivax* (2,280 parasites/μl). The patient was diagnosed with uncomplicated *P. vivax* malaria. Given that the patient had not been in endemic areas of malaria, undetected parasites in pre-transplantation blood tests, and, as mentioned, the liver donor from a malaria-endemic area in Colombia, it was concluded that the infection was donor-derived.

After the diagnosis, the patient was treated with artemether/lumefantrine at a dose of 80 mg/480 mg per day for six days. On the first day of hospitalization (also day one of treatment), the anemia worsened (hemoglobin level of 6.8 g/dl), so the patient required a transfusion of two units of red blood cells. After two days of treatment, the parasite count decreased to 480 parasites/μΙ and, on the third day of treatment, *P. vivax* parasites were not detected, hemoglobin levels were stable, and platelet count had increased. The patient showed favorable clinical evolution and was discharged on the ninth day of hospitalization. Conventional chloroquine and primaquine treatments were not prescribed for the elimination of hypnozoites as these drugs were unavailable in Colombia at the time.

Two weeks after discharge, a high replication of CMV was detected in a follow-up visit at the outpatient service. The immunosuppressive therapy was adjusted (azathioprine 100 mg once a day), and the patient started valganciclovir treatment (900 mg twice a day).

Two months after the discharge, the patient returned to the emergency service due to malaise, fever (38.5°C), and chills. Laboratory tests showed thrombocytopenia and elevated creatinine levels; the CMV-DNA test was negative, but *P. vivax* was confirmed by a thick blood smear test (8,200 parasites/μΙ). The diagnosis was a malaria relapse secondary to residual hypnozoites. The patient immediately started treatment with chloroquine (3 days) and primaquine (14 days) as they were available. On day four of treatment, the patient no longer had fever, had a standard platelet count, and the signs of acute kidney injury improved. The patient was discharged after four days with the indication to complete the primaquine regime at home.

No other complications or relapses of malaria were reported during the one-year follow-up visits. Since the liver donor came from a malaria-endemic area, the infection was attributed to donor-derived hypnozoites in the allograft.

## Case 2

A 58-year-old woman living in the department of Córdoba, a malariaendemic area of Colombia (*P. vivax* and *P. falciparum*), came to the emergency service in April 2016 with two days of malaise, fever, chills, vomiting, arthralgia (mainly in the knees), headache, and lumbar pain.

The patient had received orthotopic liver transplantation six months before due to monofocal hepatocarcinoma that met the Milan criteria and cirrhosis (Child-Pugh class B) secondary to autoimmune hepatitis.

Prior to undergoing the transplant, she had suffered from ascites, spontaneous bacterial peritonitis, esophageal varices, and hepatic encephalopathy. At the time of consultation, the patient’s immunosuppression maintenance therapy consisted of prednisolone (5 mg once a day), tacrolimus (1 mg twice a day), and mycophenolate mofetil (1,000 mg twice a day). She was also receiving nystatin (500,000 IU three times a day) and trimethoprim/ sulfamethoxazole (160/800 mg three times per week). Furthermore, the patient had suffered multiple episodes of malaria infection until the age of 17, with no new symptomatic episodes since then. Likewise, three months before her consultation, the patient had been hospitalized due to pneumonitis (without respiratory failure) secondary to varicella-zoster virus infection, treated at the time with acyclovir.

At the physical examination on admission, the patient had a pulse of 115 bpm, a respiratory rate of 18 breaths per minute, blood pressure of 118/80 mm Hg, body temperature of 38.8°C, and oxygen saturation of 95%. Likewise, no signs of splenomegaly, hepatomegaly, or neurological deficit were observed. The patient’s complete blood count and blood chemistry tests revealed hypertransaminasemia (predominantly aspartate aminotransferase - AST), mild elevation of direct bilirubin, leukopenia, mild anemia, and thrombocytopenia. The complete results of these tests are shown in table 1. CMV-DNA viral load test, dengue immunoglobulin M, and dengue NS1 antigen tests were negative. Due to radioopacities observed in the chest X-ray, a computed tomography was performed, showing no abnormal findings.

In addition, peripheral blood cultures were negative for aerobic microorganisms, but a thick blood smear revealed *P. vivax* (50,640 parasites/ μΙ). The patient was diagnosed with uncomplicated malaria and was treated with chloroquine (three days) and primaquine (14 days), according to the national guidelines for malaria management. Thick blood smears were performed daily to monitor parasite count, and on the third day of treatment, they were no longer detected. An improvement in aminotransferase levels and cytopenias was also observed. Since there were no complications, the patient was discharged on the fifth day of treatment with instructions to complete the primaquine regime at home.

Considering the timing of the malaria onset and the patient’s residence in a malaria-endemic area, we concluded that the infection was most likely of vector-borne origin; more remotely, the infection could derive from the graft, but it occurred six months post-transplantation.

### Ethical considerations

The present report was conducted in accordance with the Declaration of Helsinki. The patient’s anonymity was always maintained.

## Discussion

Malaria is a mosquito-borne disease caused by *Plasmodium* spp., of which five species are known to infect humans. *Plasmodium* spp. is transmitted by the bite of *Anopheles* mosquitoes ^3^ and is geographically limited to areas with constant high temperatures and environmental conditions that support *Anopheles* survival, reproduction, and completion of parasites’ life cycle ^4^. In 2021, an estimated 247 million malaria cases and 619,000 malaria deaths were reported worldwide, most of them in Africa (over 90% of cases). In the Americas, 597,000 cases and 334 deaths were documented, with 10% of these cases reported in Colombia ^5^.

The severe form of malaria causes fever, malaise, hematologic cytopenias, altered level of consciousness, high parasitemia, severe anemia, disseminated intravascular coagulation, and organ dysfunction such as circulatory dysfunction, shock, acute kidney injury, and respiratory failure. Additionally, although previous exposure to malaria does not lead to complete immunity, it may protect against severe forms of the disease ^6^.

In solid organ transplant recipients, the causes of malaria infection include blood transfusions, previous infection in the donor, natural transmission through *Anopheles* mosquito bite in endemic areas, and reactivation of latent hypnozoites of *P. vivax* or *P. ovale* in the donor’s liver. Regarding infection through blood transfusion, *Plasmodium* spp. can survive 24 hours in blood stored at 4°C ^7^. Malaria following liver transplantation may occur at any time, but reports suggest that it is most common within the first two months. For this reason, malaria should be one of the differential diagnoses in liver transplant recipients presenting with fever, concomitant anemia, hemolysis, thrombocytopenia, or splenomegaly especially during this time. Even if the area where the patient lives is not endemic to malaria, donors can come from endemic areas.

In the two cases reported here, malaria occurred within the first year after liver transplantation, in case 1, one month later and, in case 2, six months later. In addition, one patient had been exposed to *Plasmodium* spp. long before suffering from the liver disease that led to the transplant. In both cases, we identify the probable origin of the infection: donor-derived infection and natural transmission (mosquito-borne) in an endemic area.

Regarding clinical signs and symptoms, both patients had thrombocytopenia, anemia, hyperbilirubinemia, fever, and malaise; they also presented with hypertransaminasemia, acute kidney injury, and musculoskeletal pain. Both patients were diagnosed with uncomplicated malaria caused by *P. vivax*, and, in both cases, the treatment with chloroquine and primaquine was effective. However, due to a shortage of chloroquine and primaquine in Colombia at the time of attendance of case 1, he was initially treated with artemether/lumefantrine, resulting in a malaria relapse after two months.

Malaria can lead to liver injury through multiple mechanisms. Liver damage caused by *Plasmodium* spp. may occur due to an ischemic injury when red blood cells infected with the parasites obstruct liver sinusoids and capillaries ^8^. Hepatotoxicity induced by antimalarial drugs is also a possibility. For example, Fischer *et al*. reported a patient who died due to acute liver failure caused by antimalarial treatment with chloroquine and primaquine ^9^. Hypertransaminasemia with predominantly elevated AST levels was observed in one of our patients (case # 2). Similarly, Chiche *et al*. ^10^ and Pandey *et al*. ^11^ described cases with elevated transaminases due to malaria.

Since the first case of malaria following liver transplantation was reported by Crafa *et al*. in 1991^12^, 17 cases (including the two described here) have been reported worldwide (table 2). The main characteristics of these patients are:

**Table 2:** Reported cases of malaria following liver transplantation

Author, year	Place	Sex	Age (years	Indication ) for LT	TP	Immunosuppression regimen	Symptoms	Onset time (days)	Plasmodium species	Infection origin	Parasite count	Treatment	Malaria complications	Outcome	Other complications
Crafa *et al*., 1991 ^12^	Italy	--	45	--	--	--	Fever, neurological disorder	22	*P. falciparum*	Donor- derived	--	Quinine	--	Death	--
Talabiska *et al*., 1996 ^13^	Pennsylvania, USA	F	64	Advanced cirrhosis secondary to HCV	OLT from DD	Prednisolone, azathioprine, cyclosporine	Fevers, chills, confusion	33	*P. ovale*	Relapse	Not reported	Chloroquine	None	Survival	UTI and CMV infection
Fischer *et al*., 1999 ^9^	Germany	F	62	Advanced cirrhosis secondary to PBC	OLT from DD	Polyclonal antithymocyte globulin, cyclosporin, methylprednisolone	Constant fever	35	*P. vivax*	Donor- derived	Not reported	Chloroquine and primaquine	Acute liver failure for four weeks after starting malaria treatment (probably DILI)	Death	Acute rejection (day 7 PT), CMV infection
Chiche *et al*., 2003 ^10^	Caen, France	M	54	Advanced cirrhosis secondary to HCV	OLT from DD	Cyclosporin, mycophenolate mofetil, methylprednisolone	Initially asymptomatic Then, thrombocytopenia The patient fell into a coma	8	*P. falciparum*	Donor- derived	15%	Quinine and doxycycline	Deterioration in liver function; high levels of bilirubin requiring three months for full recovery	Survival	None
Mejía *et al*., 2006 ^14^	Bogotá, Colombia	F	46	Acute liver failure of unknown cause	OLT from DD	Methylprednisolone, azathioprine, cyclosporin, daclizumab	Fevers, chills, and anemia	22	*P. vivax*	Donor- derived	Not reported	Chloroquine and primaquine	None	Survival	Seizures and central pontine myelinolysis (day 5 PT); UTI by P. aeruginosa and E. cloacae
Menichetti *et al*., 2006 ^15^	Italy	M	64	Acute liver failure of unknown etiology	OLT from DD	Methylprednisolone and cyclosporin	Fever, anemia, acute kidney injury, and confusion	3	*P. falciparum*	Donor- derived	10%	Quinine and doxycycline	Renal replacement therapy	Survival	Right-sided heart failure and respiratory failure in immediate PT period
Rodríguez *et al*., 2007 ^16^	Santiago de Compostela, Spain	M	30	Acute liver failure secondary to HBV	OLT from DD	Not reported	Fever, chills, hypotension, pancytopenia, and hemolysis	21	*P. vivax*	Donor- derived	80%	Chloroquine and primaquine	Fever and parasitemia recurrence after two months	Survival	None
Pandey *et al*., 2008 ^11^ (case #1)	Singapore, Republic of Singapore	F	65	HCC in cirrhotic liver	OLT from LD	Basiliximab, tacrolimus, mycophenolate mofetil, everolimus; prednisolone started on day 21	Transaminase elevation, and fever	21	*P. vivax*	Not reported	0.5%	Mefloquine and primaquine	None	Survival	None
Pandey *et al*., 2008 ^11^ (case #2)	Singapore, Republic of Singapore	M	53	Advanced alcoholic cirrhosis	OLT from LD	Prednisolone, tacrolimus	Fever	11	*P. vivax*	Not reported	0.5%	Chloroquine and primaquine	None	Survival	Small-for-size liver syndrome; pneumonia, respiratory failure, mechanical ventilation
Seth *et al*., 2009 ^17^	New Delhi, India	M	30	Advanced cryptogenic cirrhosis	OLT from DD	Corticosteroid, tacrolimus, mycophenolate mofetil; methylprednisolone for acute rejection	Intermittent fever	44	*P. vivax and* *P. falciparum*	Vector borne	Not reported	Quinine, artemether, chloroquine	None	Survival	Hepatic artery thrombosis (day 2 PT); acute rejection (day 7)
Sabé *et al*., 2014 ^18^	Barcelona, Spain	--	--	--	OLT from DD	Not reported	Asymptomatic Detected due to another recipient developing symptomatic malaria	--	*P. falciparum*	Donor- derived	Negative blood smear; positive antigen test	Quinine and doxycycline	None	Survival	None
Martin- Dávila *et al*., 2018 ^19^	Madrid, Spain	M	55	--	OLT from DD	Not reported	Asymptomatic Detected due to another recipient developing symptomatic malaria (30 days PT)	30	*P. malariae and P. ovale*	Donor- derived	Negative blood smear; positive PCR	Chloroquine and primaquine	None	Survival	None
Vita *et al*., 2021 ^20^	Rome, Italy	M	2	Advanced cirrhosis secondary to biliary atresia	OLT from LD	Steroids, mycophenolate mofetil, cyclosporine	Fever and anemia	1 year	*P. falciparum*	Vector borne	10%	Artesunate, artemether/ lumefantrine	Severe anemia requiring blood transfusion	Survival	None
Shaikh *et al*., 2021 ^21^	Mumbai, India	M	38	Advanced cryptogenic cirrhosis	OLT from DD	Prednisolone, mycophenolate mofetil, tacrolimus	Fever, chills, anemia, and thrombocytopenia	7 years	*P. vivax*	Vector borne (presumed)	Not reported	Artesunate and primaquine	None	Survival	COVID-19 pneumonia, treated with dexamethasone and remdesivir
Rosso *et al*., 2021 ^22^	Cali, Colombia	M	50	Advanced cirrhosis (cause not reported)	OLT from DD	Cyclosporin	Fever, myalgia, arthralgia, and thrombocytopenia	29	*P.* *vivax*	Donor- derived	qPCR: 100.808 copies/μΙ	Chloroquine and primaquine	None	Survival	None
Case #1 in the present report, 2024	Medellín, Colombia	M	54	Advanced cirrhosis secondary to MetALD	OLT from DD	Prednisolone, azathioprine, tacrolimus	Malaise, chills, fever, thrombocytopenia	30	*P.* *vivax*	Donor- derived	2,280 par/μΙ	Artemether/ lumefantrine Chloroquine and primaquine due to relapse	Severe anemia requiring blood transfusion; splenomegaly with infarctions; relapse	Survival	Low CMV replication, mild COVID-19
Case #2 in the present report, 2024	Medellín, Colombia	F	58	Cirrhosis (due to autoimmune hepatitis) and hepatocarcinoma	OLT from DD	Prednisolone, mycophenolate mofetil, tacrolimus	Fever, chills, vomiting, arthralgia, headache, lumbar pain, thrombocytopenia	6 months	*P.* *vivax*	Vector borne	50,640 par/μΙ	Chloroquine and primaquine	None	Survival	None

CMV: Cytomegalovirus; DD: Deceased donor; DILI: Drug-induced liver injury; HBV: Hepatitis B virus; HCV: Hepatitis C virus; LD: Living donor; LT: Liver transplantation; MetALD: Metabolic dysfunction-associated alcohol-related liver disease; OLT: Orthotopic liver transplantation; PBC: Primary biliary cholangitis; PCR: Polymerase chain reaction; TP: Type of transplantation; PT: Post-transplantation; UTI: Urinary tract infection

*Demographics*: Only one case was reported in the pediatric population (a two-year-old boy) ^20^. The remaining patients ranged from 30 to 65 years. Nine cases were men, five were women, and two did not report the sex.

*Type of transplant and time of malaria symptoms presentation*: Most cases (n = 13) corresponded to a deceased donor liver transplantation, three were living-donor liver transplant, and one did not report information about the type of transplant. On the other hand, malaria symptoms presented within 35 days following liver transplantation in 12 cases, while in the remaining ones, the onset of symptoms was reported on day 42, month six, and one year after liver transplantation; one case did not report this information.

*Mechanism of infection*: Donor-derived transmission was reported in ten cases, natural transmission was documented in four, and disease reactivation was suspected in one ^13^; in two cases they did not report information about the origin of the infection.

*Symptoms*: Two cases were asymptomatic. Malaria was diagnosed after confirming donor-derived transmission in recipients of other organs from the same donors (heart and kidney) ^18^^,^^19^. In symptomatic cases, fever was documented in all but one, and anemia and thrombocytopenia were very common.

*Complications and outcomes*: Three patients had serious complications, such as acute liver failure, deep coma, and acute kidney injury that required renal replacement therapy. Two deaths were reported: one was not directly related to malaria complications (acute liver failure secondary to malaria treatment) ^9^, and the other was due to infection with *P. falciparum*^12^.

*Plasmodium infective species*: *P. vivax* was reported in ten cases, *P. falciparum* in six, *P. malariae* in one, and *P. ovale* in two. Three cases documented concomitant infection by two species.

*Geographic distribution*: All cases were reported in Europe, the Americas, and Asia. Despite most cases of malaria occur in Africa, no malaria post transplantation reports have been documented there, probably because of its low solid organ transplantation rate ^23^.

Multiple recipients of solid organs from a single donor have been reported to develop malaria infection ^9^^,^^10^^,^^15^^,^^16^^,^^18^^,^^19^. In some of these reports, the liver recipients with malaria were asymptomatic, and the outcome was favorable after completing treatment ^18^. This scenario emphasizes the importance of urgent notification of malaria cases in solid organ recipients to the transplant networks and health care system authorities, to promote rapid detection and timely treatment of asymptomatic cases.

Transplantation of organs from donors living in malaria-endemic regions is a potential source of *Plasmodium* infection in non-endemic areas. Thus, the donor’s place of origin defines the probability of parasitemia. In a systematic review and meta-analysis about malaria prevalence among immigrants in Europe, North America, and Oceania, the authors reported a low prevalence in migrants from Asia and Latin America, moderate in those from West Africa, and high in those from Central Africa ^24^. These studies could help implement rational and focused screening strategies for detecting *Plasmodium* spp. in donors and recipients, especially when the donor has a high epidemiological probability of being infected with these parasites. In malaria-endemic countries with multiple potential regions of transmission, such as Colombia, the assessment of transplant recipients presenting with fever must consider the place of origin of the donor and the recipient.

Detection of *Plasmodium* spp. in organ donors from malaria-endemic countries depends on local prevalence. Africa is likely the only continent where screening all patients is cost-effective. In the case of Latin America and Asia, screening should be performed only in those coming from areas where parasite transmission is possible ^25^. Blood smears, antibody serology tests, rapid antigen diagnostic tests, and nucleic acid amplification tests are used to detect malaria. So far, the best method is nucleic acid amplification tests since they detect *Plasmodium* spp. in cases with low parasitemia, as those of asymptomatic patients, particularly in individuals who have been exposed to the parasite; this technique has a higher sensitivity compared to blood smear and rapid antigen detection tests ^6^.

Solid organ transplantation from donors with active malaria infection should be avoided ^2^. In the case of donors with a treated infection, the safe time between treatment completion and the transplantation has not yet been determined. In high-risk scenarios, like those involving potential exposure, preventive treatment during the post-transplant period is recommended. On the other hand, liver transplant patients who live in or travel to areas where *Plasmodium* spp. circulates should take preventive measures, such as using barrier methods (clothes, mosquito nets), and insecticides. Pharmacological prophylaxis is also an option to be considered ^2^.

Outcomes in liver transplant recipients with malaria seem to be better than initially thought. Two deaths have been reported, one of those directly caused by malaria ^12^ and the other by its treatment ^9^. The best outcomes are prone to depend on early detection, timely start of antiparasitic treatment, and adequate supportive management. Full recovery from malaria (i.e., complete clearance of the parasites from the body) could also benefit from low-dose immunosuppressive therapies used in liver transplantation.

Finally, provided that in cases in which malaria infection by *P. falciparum* after liver transplantation was confirmed (5 out of 17 cases) (table 2), serious complications were reported including one death, the presence of this species in these patients requires special surveillance.

## Conclusion

Malaria is a potential complication in liver recipients, especially within the first two months after receiving the transplantation. Malaria screening must be considered in solid organ transplant donors or recipients from endemic areas where this infection is highly prevalent. Likewise, malaria should be suspected in solid organ transplantation recipients presenting with fever, unexplained anemia, and thrombocytopenia in endemic and non-endemic areas with migrant donors.

It seems that the clinical course and manifestations of this infection in liver transplant patients do not differ significantly from those described in immunocompetent individuals. This similarity is probably related to early diagnosis and timely treatment, which are essential for achieving a favorable outcome. In addition, *P. falciparum* infections require special monitoring and caution. It is worth noting that we need more research on epidemiology, detection, and prevention of malaria in liver transplant patients.
